# Impaired sensitivity to thyroid hormone is associated with developing non-alcoholic fatty liver disease in euthyroid diabetic subjects

**DOI:** 10.3389/fendo.2024.1450049

**Published:** 2024-12-18

**Authors:** Xiaowen Zhang, Jie Liu, Qian Wang, Chen Han, Yu Yan, Xinyue Xiang, Shanmei Shen, Wenhuan Feng

**Affiliations:** ^1^ Department of Endocrinology and Metabolism, Endocrine and Metabolic Disease Medical Center, Nanjing Drum Tower Hospital, The Affiliated Hospital to Nanjing University Medical School, Nanjing, China; ^2^ Branch of National Clinical Research Centre for Metabolic Diseases, Nanjing, China; ^3^ Department of Endocrinology and Metabolism, Nanjing Drum Tower Hospital, Chinese Academy of Medical Science and Peking Union Medical College, Nanjing, China; ^4^ Department of Endocrinology and Metabolism, Nanjing Drum Tower Hospital, Clinical Medical College of Southeast University, Nanjing, China; ^5^ Department of General Surgery, Nanjing Drum Tower Hospital, Clinical Medical College of Southeast University, Nanjing, China; ^6^ Department of Endocrinology, Endocrine and Metabolic Disease Medical Center, Nanjing Drum Tower Hospital Clinical College of Nanjing University of Chinese Medicine, Nanjing, China

**Keywords:** fibrosis score, non-alcoholic fatty liver disease, thyroid hormone sensitivity, type 2 diabetes, health screening

## Abstract

**Background and aims:**

Acquired resistance to thyroid hormone appears to exist in the general population. We aimed to evaluate the association between indices of thyroid hormone sensitivity and non-alcoholic fatty liver disease (NAFLD), and made stratified analyses by diabetic status.

**Methods:**

We included 26,413 participants from a health screening program and 8,246 hospitalized patients with type 2 diabetes. Thyroid Feedback Quantile-based Index (TFQI), thyroid stimulating hormone index (TSHI) and thyrotroph thyroxine resistance index (TT4RI) were calculated. Advanced fibrosis risk was determined using the FIB-4 score. Multivariate logistic regression analysis was performed.

**Results:**

TFQI was associated with an increased risk of NAFLD in patients with diabetes (fourth quartile vs. first quartile: odds ratio [OR]=1.39 and 1.82 in hospitalized and non-hospitalized patients, respectively, both P<0.001) but not non-diabetic participants (OR=0.94, P=0.40). Further adjustment for the homeostasis model assessment of insulin resistance generated similar findings in diabetes (OR=1.27, P=0.025). The TFQI-associated NAFLD risk increase in diabetic patients was confined to NAFLD with low probability of advanced fibrosis (OR 1.42, P=0.001), but not those with intermediate-to-high probability (OR=0.86, P=0.23). Also, TFQI was associated with a significantly lower risk for advanced fibrosis in the diabetic at-risk patients (OR=0.62, P=0.005) but not those non-diabetic at-risk participants, independent of the presence of NAFLD. The association was less significant for TT4RI and TSHI.

**Conclusions:**

Impaired sensitivity to thyroid hormone was associated with an increased risk of developing NAFLD but a reduced risk of advanced fibrosis limited to diabetic individuals. Our findings suggest stratified studies of NAFLD based on diabetic status are needed in the future.

## Introduction

The prevalence of non-alcoholic fatty liver disease (NAFLD) has been growing rapidly over the last four decades, affecting over 25% of adults worldwide now (1). NAFLD has become one of the leading causes of chronic liver disease, significantly contributing to end-stage liver disease and the demand for liver transplantation, as well as increasing risks for hepatic cirrhosis, liver cancer, and cardiovascular diseases (2). NAFLD is similarly common in Asia (3). Although Asian patients tend to have less severe liver histology and better clinical outcomes, this huge number of affected patients still gives rise to considerable cases of hepatocellular carcinoma (3). Our understanding of the risk factors of NAFLD has improved, but the still increasing prevalence promotes further in-depth investigation of other modifiable risk factors for NAFLD.

NAFLD is a complex disease, and its pathophysiology involves diverse aspects, including disturbances in glucose and lipid metabolism, lipotoxicity, insulin resistance, chronic inflammation, and disruptions in intestinal function and gut microbiome composition, etc (4). Thyroid hormones (TH) are essential for maintaining energy homeostasis and are actively involved in a range of metabolic pathways, including glucose metabolism (regulating gluconeogenesis and glucose uptake), lipid metabolism (promoting lipolysis and fatty acid oxidation), cholesterol metabolism (modulating synthesis and clearance), and the basal metabolic rate (5). The association between TH, TH metabolites, and the presence and severity of NAFLD has been investigated in a number of observational studies, but with controversial findings (6). Some studies showed that the presence of hypothyroidism or subclinical hypothyroidism was associated with a significantly increased risk of NAFLD (7), but others failed to reveal a significant association (8), or even showed an opposite association (9).

Theoretically, an acquired resistance to thyroid hormones (TH, specifically T3 and T4), presented as a higher level of thyroid stimulating hormone (TSH) as well as TH, might help explain these conflicting results. A new index of resistance to TH, named Thyroid Feedback Quantile-based Index (TFQI), was developed by Dr. Laclaustra and colleagues (10). TFQI focused on deviations of the average pituitary response (inhibition) to TH in the general population, and has been shown in several studies to be associated with metabolic syndrome, hypertension (11), cardiovascular disease risk (12), diabetes and diabetes-related mortality (10, 13), in euthyroid subjects or subjects with subclinical hypothyroidism.

One study with participants from a health examination center showed no association between TH sensitivity index TFQI and NAFLD (odds ratio [OR] 0.9, 95% CI 0.75-1.09) (14), but this finding was limited by its modest-to-moderate sample size. Considerable heterogeneity exists in the patient phenotype in individuals with NAFLD (15). Notably, the prevalence of NAFLD and advanced liver fibrosis increase in patients with diabetes and prediabetes, and vice versa (16). The bidirectional and complex interaction between NAFLD and type 2 diabetes involves key mechanisms, such as insulin resistance and subclinical inflammation, that promote metabolic dysregulation in both conditions (15)., This evidence supports the idea that stratifying patients with NAFLD by diabetic status might improve the diagnosis of NAFLD and prediction of its progression (16). As such, recent clinical care pathway recommends that patients with type 2 diabetes screening for NAFLD with advanced fibrosis (17).

Therefore, in this study, we first explored whether TH sensitivity indices, including TFQI, are associated with the risk of NAFLD in a large sample of community-dwelling euthyroid subjects, conducted stratified analyses by diabetic status, and then validated the findings of diabetic population in another large sample of hospitalized type 2 diabetic patients.

## Methods

### Study populations

**Figure 1:**
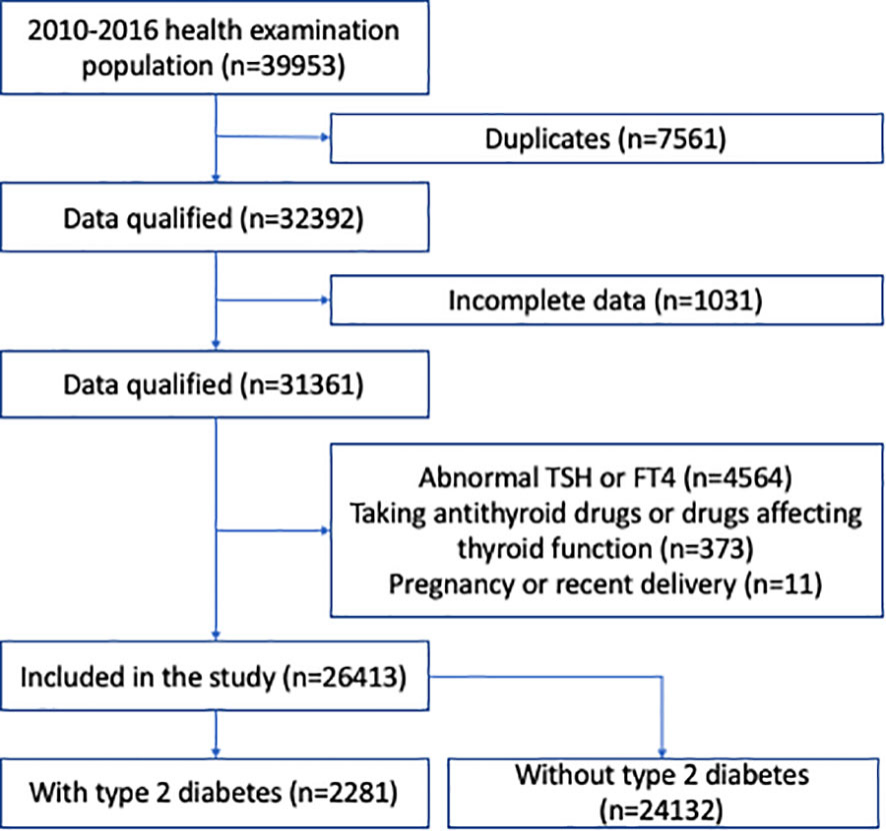
Flow chart of the study participants in the health examination population.

**Figure 2:**
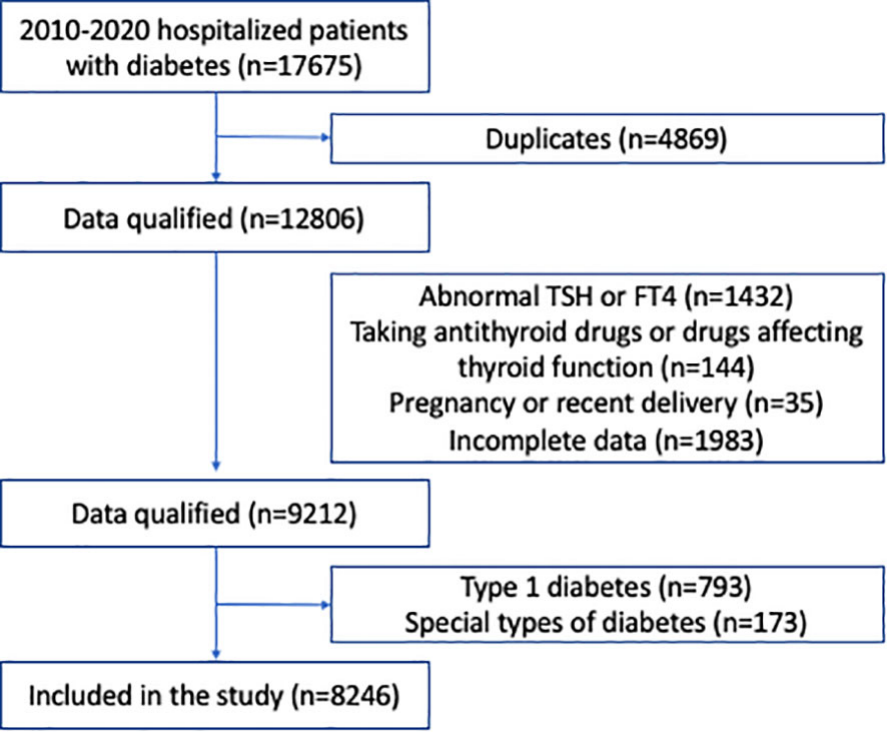
Flow chart of the hospitalized patients with type 2 diabetes.

Two independent cohorts were assessed in our study. The first cohort consisted of all participants in a health screening program by a large health-care institution affiliated to Drum Tower Hospital, from January 2010 to December 2016. The second cohort consisted of all type 2 diabetic patients who were hospitalized in the Department of Endocrinology and Metabolism, the Affiliated Drum Tower Hospital, Nanjing University School of Medicine, from January 2010 to December 2020. In both cohorts, we included participants >18 years of age, who had normal thyroid function based on thyroid stimulating hormone (TSH, 0.27-4.2 mIU/L) and free thyroxine (fT4, 12.0-22.0 pmol/L). We excluded those who were concomitantly prescribed antithyroid drugs, as well as drugs that might affect thyroid function, such as amiodarone or corticosteroids. We also excluded participants previously diagnosed with thyroid disorders, those who were pregnant or had recent delivery, and those with incomplete data. In the cohort of hospitalized type 2 diabetic patients, those with type 1 diabetes or special types of diabetes were excluded. The screening process is shown in **Figures 1**, **2**. This study protocol was approved by the Ethics Committee of Nanjing Drum Tower Hospital Institutional Review Board, and carried out in accordance with the Declaration of Helsinki. Consent was waived for this retrospective study.

### Demographic data and laboratory assays

Baseline characteristics, including age, gender, smoking status, alcohol consumption, medical history, medication usage, and other sociodemographic variables were obtained by questionnaire. Weight, height, and blood pressure were measured using standard methods and body mass index (BMI) was calculated as weight (kg) divided by the square of height (m). Blood samples were collected from all participants after overnight fasting for at least 8 hours. Serum TSH, free triiodothyronine (FT3), FT4, thyroid autoantibodies (thyroid peroxidase antibody [TPOAb]), and thyroglobulin antibody [TgAb]) concentrations were detected by electrochemical luminescence assays with Cobas Eless 601 (Roche). The reference ranges of TSH, FT3, FT4, TPOAb and TgAb were 0.27–4.2 mIU/L, 3.1-6.8 pmol/L, 12.0-22.0 pmol/L, 0–34 IU/mL, and 0–115 IU/mL respectively, as provided by the manufacturer. Intra and inter-assay coefficients of variability (CVs) were 1.2% and 3.4% for FT4, and 1.5% and 2.9% for TSH, respectively.

Fasting plasma glucose (FPG) was assessed by the glucose oxidase method, and plasma insulin concentrations were tested by radioimmunoassay. Insulin resistance was determined with the homeostasis model assessment of insulin resistance (HOMA-IR), which was calculated as (fasting glucose [mmol/L]) × (fasting insulin [μU/mL])/22.5. Glycated hemoglobin (HbA1c) concentrations were measured by high-performance liquid chromatography. Serum high-density lipoprotein cholesterol (HDL-C) levels were tested using standard enzymatic methods (Kyowa Medex Co., Ltd. Tokyo, Japan), and serum low-density lipoprotein cholesterol (LDL-C) levels were measured using selective melt method (Kyowa Medex Co., Ltd. Tokyo, Japan). Serum alanine aminotransferase (ALT) and aspartate aminotransferase (AST) levels were measured, and an elevated liver enzyme level was defined as a serum concentration of either AST ≥40 U/L or ALT ≥40 U/L.

### Assessment of hepatic steatosis and NAFLD severity

Abdominal ultrasound imaging was performed by experienced radiologists who were unaware of the study aims, using iU22 xMatrix (Philips Medical Systems, Cleveland, OH, USA). Fatty liver was diagnosed using standard criteria, including a diffuse increase in fine echoes in the liver parenchyma in comparison with the kidney or spleen, deep beam attenuation, and bright vessel walls (18).

Fibrosis risk for both cohorts was determined using the FIB-4 score, which was calculated using the following formula: FIB-4 = (age [years] ×AST [U/L])/(platelet count [× 10^9^/L] ×ALT [U/L]^1/2^). A low-, intermediate- and high-probability for advanced fibrosis was defined as a FIB-4 score of <1.3 for participants <65 years old (or <2.0 for those ≥65 years old), 1.3 to 2.67 for participants <65 years old (or 2.0 to 2.67 for those ≥65 years old), and >2.67, respectively (17, 19).

### Indices of TH sensitivity

Three indices were calculated to assess central TH sensitivity. The TSH index (TSHI) was calculated as ln TSH (mIU/L) + 0.1345 × FT4 (pmol/L), the TSH T4 resistance index (TT4RI) as TSH (mIU/L) × FT4 (pmol/L), and TFQI as cdf FT4- (1-cdf TSH), as previously reported (10). A positive TFQI indicates poor TH sensitivity, and a negative value indicates good TH sensitivity.

### Definitions

Type 2 diabetes was defined according to ADA guidelines as hemoglobin A1c (HbA1c) ≥6.5% and/or fasting plasma glucose ≥7.0 mmol/L, or a self-reported history of diabetes. Hypertension was defined as systolic blood pressure ≥140 mmHg or diastolic blood pressure ≥90 mmHg, and/or current use of antihypertensive medications. Chronic kidney disease (CKD) was defined as an estimated glomerular filtration rate (eGFR) <60 ml/min/1.73 m^2^. NAFLD was defined based on ultrasound evidence of fatty liver, in the absence of other causes of chronic liver disease or excessive alcohol consumption (>30 g/day for males and, >20 g/day for females).

### Statistical analyses

Analyses were conductedusing SPSS (version 26.0). Two-tailed P values <0.05 were considered significant. Continuous variables were presented as means ± standard deviation or median (interquartile range), while categorical variables were presented as numbers with percentages. Differences between subgroups were analyzed with one-way analysis of variance or Mann–Whitney U-test for continuous variables, and χ^ 2^ test for categorical variables. OR with 95% confidence intervals (CIs) across quartiles were calculated with binary logistic regression analysis, to assess the association between each TH sensitivity index with NAFLD development, using the lowest quartile as reference. All analyses were adjusted for potential confounders, and adjustments were different between the health examination population and hospitalized type 2 diabetic patients. In the examination cohort, model 1 was adjusted for age, sex; model 2 further adjusted for BMI; model 3 further adjusted for hypertension, HbA1c, triglyceride, and total cholesterol. In the hospitalized diabetic cohort, model 1 was adjusted for age, sex; model 2 further adjusted for smoking status, alcohol habitus (classified as drinker vs. non-drinker due to lack of quantitative intake data), and BMI; model 3 further adjusted for duration of diabetes, family history for diabetes, hypertension, HbA1c, triglyceride, total cholesterol, and medications; model 4 further adjusted for HOMA-IR. Significance for trend was tested with each index quartile ordinal as a continuous variable.

To investigate whether each TH sensitivity index could predict the risk of advanced liver fibrosis in participants at risk, multivariate logistic regression analysis was performed, using the adjustment models with most variables in the previous analysis of NAFLD, such as age, gender, BMI, and metabolic risk factors. In the health examination cohort, three types of at-risk participants, i.e., those with two or more metabolic risk factors, with type 2 diabetes, or with steatosis on imaging or elevated aminotransferases, were included in the analysis (17); while in the hospitalized diabetic cohort, all type 2 diabetic patients were included in the analysis because they were all considered at-risk.

## Results

### Baseline characteristics and association analysis of NAFLD in the whole health-examination cohort

We identified 32,392 subjects for health examination from January 1, 2010 to December 31, 2016 after removing duplicates; 5,979 subjects were excluded according to the exclusion criteria (**Figure 1**). Finally, 26,413 participants were included for analysis. Among these participants, 15,247 (57.7%) were male, 2,281 (8.6%) had type 2 diabetes, 5,481 (20.8%) had hypertension, and 5,640 (21.4%) had NAFLD (**Supplementary Table S1**). The mean age was 48.3 years, and BMI was 24.2 kg/m^2^ (**Supplementary Table S1**).

We first investigated the independent risk of NAFLD according to the TH sensitivity indices and diabetes status in the overall health-examination population. As shown in **Table 1**, TFQI was not significantly associated with NAFLD; however, diabetic status showed a significant effect on NAFLD development (OR 2.47, 95% CI 2.12-2.88, P <0.001) even after adjustment for age, sex, BMI, hypertension, HbA1c, triglycerides, and total cholesterol. Similar observations were found for TT4RI and TSHI (**Table 1**).

**Table 1:** Associations between thyroid hormone sensitivity, diabetics status and NAFLD in the overall health-examination population.

		Model 1		Model 2		Model 3	
		OR (95% CI)	P value	OR (95% CI)	P value	OR (95% CI)	P value
TFQI	Quartile 1	1.00 (Reference)		1.00 (Reference)		1.00 (Reference)	
	Quartile 2	0.99(0.91, 1.08)	0.894	1.00 (0.91, 1.1)	0.933	1.09 (0.97, 1.23)	0.139
	Quartile 3	1.00 (0.92, 1.09)	0.99	1.02 (0.93, 1.12)	0.685	1.15 (1.02, 1.29)	0.022
	Quartile 4	1.04 (0.95, 1.27)	0.426	1.05 (0.96, 1.16)	0.276	1.06 (0.94, 1.19)	0.372
	P value for trend		0.428		0.257		0.261
Diabetes	Yes/no					2.47 (2.12, 2.88)	<0.001
TT4RI	Quartile 1	1.00 (Reference)		1.00 (Reference)		1.00 (Reference)	
	Quartile 2	1.04 (0.96, 1.14)	0.345	1.06 (0.97, 1.17)	0.214	1.05 (0.94, 1.19)	0.394
	Quartile 3	1.02 (0.94, 1.12)	0.598	1.03 (0.93, 1.13)	0.607	1.04 (0.92, 1.17)	0.529
	Quartile 4	1.06 (0.97, 1.15)	0.221	1.06 (0.96, 1.16)	0.251	1.06 (0.94, 1.19)	0.383
	P value for trend		0.393		0.391		0.454
Diabetes	Yes/no					2.47 (2.12, 2.88)	<0.001
TSHI	Quartile 1	1.00 (Reference)		1.00 (Reference)		1.00 (Reference)	
	Quartile 2	1.02 (0.93, 1.11)	0.684	1.04 (0.94, 1.14)	0.47	1.14 (1.01, 1.29)	0.032
	Quartile 3	1.05 (0.96, 1.14)	0.264	1.05 (0.95, 1.15)	0.325	1.14 (1.01, 1.29)	0.028
	Quartile 4	1.02 (0.94, 1.11)	0.674	1.04 (0.94, 1.14)	0.481	1.05 (0.93, 1.18)	0.439
	P value for trend		0.532		0.451		0.448
Diabetes	Yes/no					2.47 (2.12, 2.88)	<0.001

Model 1 is adjusted for age, sex; model 2 is adjusted for age, sex, and BMI; model 3 is adjusted for age, sex, BMI, hypertension, HbA1c, triglyceride, and total cholesterol.

OR, odds ratio; TFQI, thyroid feedback quantile-based index; TSHI, TSH index; TT4RI, Thyrotropin T4 resistance index.

### Baseline characteristics and association analysis of NAFLD in the health-examination cohort stratified by diabetic status

We then asked whether there were differences in characteristics and effects of TH sensitivity indices between participants with and without type 2 diabetes. The baseline characteristics were presented by diabetes status (**Supplementary Table S1**). Male, NAFLD, glycemic parameters, total cholesterol, triglycerides, LDL-C, liver enzymes, TSH and FT4 levels were higher in the type 2 diabetic group than in the non-diabetic group, while HDL-C, platelet count, and FT3 levels were higher in the non-diabetic group than in the type 2 diabetic group (**Supplementary Table S1**).

The remarkable differences in TH levels between participants with and without diabetes led us to perform stratified analysis of these two groups. Baseline characteristics of 24,132 non-diabetic participants were provided according to TFQI quartiles (**Supplementary Table S2**). With higher TFQI quartiles, the HbA1c level of the population was progressively lower, while albumin level was progressively higher. However, no difference was detected in age, sex, blood pressure, lipids, hypertension, or NAFLD incidence (Q 1 to Q4: 21.0%, 20.2%, 19.5%, 20.0%, respectively, P =0.23) between different TFQI categories (**Supplementary Table S2**). Logistic regression analysis also did not reveal a significant association between TFQI, TT4RI, TSHI and NAFLD in all models adjusted (**Table 2**).

**Table 2:** Associations between thyroid hormone sensitivity and NAFLD in the non-diabetic health examination population.

		Model 1		Model 2		Model 3	
		OR (95% CI)	P value	OR (95% CI)	P value	OR (95% CI)	P value
TFQI	Quartile 1	1.00 (Reference)		1.00 (Reference)		1.00 (Reference)	
	Quartile 2	0.96 (0.87, 1.05)	0.329	0.96 (0.87, 1.06)	0.431	1.05 (0.92, 1.2)	0.465
	Quartile 3	0.9 (0.82, 0.99)	0.022	0.9 (0.81, 1.0)	0.04	1.0 (0.88, 1.14)	0.974
	Quartile 4	0.95 (0.87, 1.04)	0.243	0.97 (0.87, 1.07)	0.501	0.94 (0.83, 1.08)	0.395
	P value for trend		0.124		0.29		0.306
TT4RI	Quartile 1	1.00 (Reference)		1.00 (Reference)		1.00 (Reference)	
	Quartile 2	1.04 (0.95, 1.14)	0.446	1.05 (0.95, 1.17)	0.305	1.06 (0.93, 1.21)	0.424
	Quartile 3	0.99 (0.9, 1.09)	0.989	0.99 (0.9, 1.1)	0.887	1.02 (0.89, 1.16)	0.845
	Quartile 4	1.01 (0.92, 1.1)	0.901	1.01 (0.91, 1.12)	0.888	1.0 (0.998, 1.005)	0.418
	P value for trend		0.842		0.816		0.855
TSHI	Quartile 1	1.00 (Reference)		1.00 (Reference)		1.00 (Reference)	
	Quartile 2	1.0 (0.91, 1.09)	0.928	1.02 (0.92, 1.13)	0.753	1.14 (0.995, 1.29)	0.059
	Quartile 3	0.98 (0.9, 1.08)	0.722	0.97 (0.88, 1.08)	0.587	1.06 (0.93, 1.2)	0.414
	Quartile 4	0.95 (0.86, 1.04)	0.237	0.97 (0.87, 1.07)	0.502	0.96 (0.84, 1.1)	0.589
	P value for trend		0.23		0.365		0.41

Model 1 is adjusted for age, sex; model 2 is adjusted for age, sex, and BMI; model 3 is adjusted for age, sex, BMI, hypertension, HbA1c, triglyceride, and total cholesterol.

OR, odds ratio; TFQI, thyroid feedback quantile-based index; TSHI, TSH index; TT4RI, Thyrotropin T4 resistance index.

In 2,281 diabetic individuals from the health-examination cohort, multivariate analysis showed that a higher TFQI value was associated with a significantly higher risk for NAFLD (Q2 vs. Q1: OR 1.34, 95% CI 0.996-1.81, P =0.053; Q3 vs. Q1: OR 2.12, 95% CI 1.59-2.82, P <0.001; Q4 vs. Q1: OR 1.82, 95% CI 1.36-2.42, P <0.001; P for trend <0.001; **Supplementary Table S3**), after adjusting for age, sex, BMI, hypertension, HbA1c, triglycerides, and total cholesterol. Similar associations were detected for TT4RI and TSHI (P for trend =0.031 and =0.001 respectively), although the magnitude of association was weaker compared to TFQI.

### Baseline characteristics and association analysis of NAFLD in the hospitalized type 2 diabetic cohort

Now we have shown a significant association between TH resistance indices and NAFLD in diabetic participants in the health-examination cohort, but many potential confounders were not adjusted in this cohort, such as the duration of diabetes and HOMA-IR. To confirm these findings, we performed an analysis of independent cohort of patients who were hospitalized for type 2 diabetes.

We identified 12,806 hospitalized subjects with type 2 diabetes from January 1, 2010 to December 31, 2020 after removing duplicates; 4,560 subjects were further excluded according to the exclusion criteria (**Figure 2**). Finally, 8,246 patients were included for analysis. Among these patients, 5,014 (60.8%) were male, 2,946 (35.7%) had hyperlipidemia, 4,616 (56.0%) had hypertension, 798 (9.7%) had CKD, and 3,838 (46.5%) had NAFLD (**Table 3**). The mean age was 56.96 years, BMI was 25.5, HbA1c level was 8.89%; the median duration of diabetes was 7.0 years (**Table 3**).

**Table 3:** Baseline characteristics of hospitalized type 2 diabetic patients according to TFQI categories.

	Total (-0.98, 0.94)	Quartile 1 (-0.98, -0.23)	Quartile 2 (-0.23, 0.02)	Quartile 3 (0.02, 0.29)	Quartile 4 (0.29, 0.94)	P value
N	8246	2061	2062	2062	2061	
Age (ys)	56.96 ± 14.12	57.44 ± 13.6	57.64 ± 13.81	56.71 ± 14.04	56.05 ± 14.95	<0.001
Sex, male	5014 (60.8%)	1270 (61.6%)	1217 (59.0%)	1259 (61.1%)	1268 (61.5%)	0.28
BMI	25.51 ± 4.67	27.25 ± 4.76	25.36 ± 4.46	25.58 ± 4.55	25.82 ± 4.89	0.018
Duration of diabetes (ys)	7 (0, 50)	7 (0, 50)	8 (0, 40)	7 (0, 40)	7 (0, 50)	0.004
Smoking	2195 (26.6%)	572 (27.8%)	520 (25.2%)	568 (27.5%)	535 (26.0%)	0.187
Alcohol habitus	1331 (15.9%)	331 (16.1%)	319 (15.5%)	315 (15.3%)	346 (16.8%)	0.547
SBP (mmHg)	136.07 ± 19.29	134.41 ± 19.32	136.12 ± 19.08	135.76 ± 19.13	137.98 ± 19.47	<0.001
HbA1c (%)	8.89 ± 2.29	8.96 ± 2.35	8.85 ± 2.29	8.78 ± 2.23	8.98 ± 2.29	0.027
FPG (mmol/L)	7.67 (1.67, 29.67)	7.5 (1.67, 28.38)	7.61 (2.59, 20.12)	7.61 (2.05, 29.67)	7.99 (1.96, 21.57)	<0.001
HOMA-IR	2.55 (0.02, 577.78)	2.49 (0.02, 577.78)	2.49 (0.03, 223.38)	2.55 (0.03, 275.33)	2.66 (0.04, 176.87)	0.197
TG (mmol/L)	1.38 (0, 50)	1.32 (0, 33.54)	1.41 (0, 37.96)	1.41 (0, 27.12)	1.41 (0, 50)	<0.001
TC (mmol/L)	4.53 ± 1.17	4.52 ± 1.25	4.56 ± 1.17	4.51 ± 1.09	4.51 ± 1.18	0.607
LDL-C (mmol/L)	2.55 ± 0.88	2.53 ± 0.87	2.58 ± 0.91	2.55 ± 0.86	2.56 ± 0.87	0.419
HDL-C (mmol/L)	1.08 ± 0.34	1.09 ± 0.36	1.08 ± 0.34	1.08 ± 0.32	1.08 ± 0.33	0.855
ALT (IU/L)	29.7 ± 47.8	28.6 ± 73.3	28.0 ± 27.7	30.3 ± 42.8	31.9 ± 33.7	0.042
AST (IU/L)	24.6 ± 37.3	24.0 ± 57.2	23.9 ± 21.9	24.6 ± 36.5	25.8 ± 22.0	0.371
Platelet count (×10^9^/L)	203.7 ± 62.6	199.6 ± 64.1	201.7 ± 62.3	204.5 ± 61.0	208.8 ± 62.6	<0.001
Albumin (g/L)	41.1 ± 3.5	40.3 ± 3.6	40.9 ± 3.4	41.5 ± 3.4	41.9 ± 3.3	<0.001
TSH (pmol/L)	1.92 ± 0.9	1.17 ± 0.43	1.79 ± 0.82	2.15 ± 0.89	2.57 ± 0.71	<0.001
FT3 (pmol/L)	4.42 ± 0.76	4.28 ± 0.77	4.36 ± 0.74	4.48 ± 0.78	4.57 ± 0.71	<0.001
FT4 (pmol/L)	17.11 ± 2.21	15.44 ± 1.45	16.43 ± 2.14	17.54 ± 1.95	19.04 ± 1.4	<0.001
TGAB (mIU/L)	11 (9.99, 4000.01)	10.9 (9.99, 4000)	11.1 (9.99, 4000.01)	11.03 (9.99, 3896)	11.1 (9.99, 4000.01)	0.288
TPOAB (mIU/L)	12.8 (4.99, 600.01)	11.72 (4.99, 600.01)	12.42 (4.99, 600.01)	12.99 (4.99, 600.01)	13.85 (4.99, 600.01)	<0.001
NAFLD	3838 (46.5%)	837 (40.6%)	929 (45.1%)	1003 (48.6%)	1069 (51.9%)	<0.0001
Hypertension	4616 (56.0%)	1050 (50.9%)	1183 (57.4%)	1154 (56.0%)	1229 (59.6%)	<0.0001
Hyperlipidemia	2946 (35.7%)	685 (33.2%)	780 (37.8%)	736 (35.7%)	745 (36.1%)	0.021
CKD	798 (9.7%)	209 (10.1%)	216 (10.5%)	177 (8.6%)	196 (9.5%)	0.178
Medications						
Metformin	2713 (32.9%)	625 (30.3%)	623 (30.2%)	713 (34.6%)	752 (36.5%)	<0.0001
Acarbose	1077 (13.1%)	364 (17.6%)	325 (15.7%)	252 (12.2%)	136 (6.6%)	<0.0001
DPP4 inhibitors	583 (7.1%)	135 (6.6%)	100 (4.8%)	172 (8.3%)	176 (8.5%)	<0.0001
Insulin	4921 (59.7%)	1234 (59.9%)	1271 (61.6%)	1200 (58.2%)	1216 (59.0%)	0.21
Statins	2173 (26.4%)	497 (24.1%)	572 (27.7%)	546 (26.5%)	558 (27.1%)	0.047
ACEI/ARB	1806 (21.9%)	386 (18.7%)	463 (22.4%)	457 (22.1%)	501 (24.3%)	0.0002
β-blockers	887 (10.8%)	183 (8.9%)	220 (10.7%)	232 (11.3%)	252 (12.2%)	0.005
Antiplatelet therapy	1305 (15.8%)	321 (15.6%)	333 (16.1%)	318 (15.4%)	333 (16.2%)	0.878

Continuous variables are presented as mean ± standard deviation (SD) or median (interquartile range). Categorical variables are presented as number (percentage).

ACEI, angiotensin-converting enzyme inhibitors; ALT, alanine transaminase; AST, aspartate aminotransferase; ARB, angiotensin receptor blockers; BMI, body mass index; CKD, chronic kidney disease; DPP4, dipeptidyl peptidase IV; FPG, fasting plasma glucose; FT3, free triiodothyronine; FT4, free thyroxine; HDL-C, high-density lipoprotein cholesterol; HOMA-IR, Homeostasis Model Assessment Index for Insulin Resistance; LDL-C, low-density lipoprotein cholesterol; NAFLD, non-alcoholic fatty liver disease; SBP, systolic blood pressure; TC, total cholesterol; TFQI, thyroid feedback quantile-based index; TG, triglycerides; TgAb, thyroglobulin antibody; TPOAb, thyroid peroxidase antibody; TSH, thyroid stimulating hormone.

With higher TFQI quartiles, the age, BMI of the population were lower, while systolic blood pressure, FPG level, triglycerides, platelet count, albumin level were higher, as well as ALT level and the percentage of patients with NAFLD and hypertension. Patients with higher TFQI quartiles tended to receive more metformin, dipeptidyl peptidase IV (DPP4) inhibitors, angiotensin-converting enzyme inhibitors (ACEI) or angiotensin receptor blockers (ARB), and β-blockers, but received less acarbose treatment (**Table 3**).

Multivariate analysis showed that a higher TFQI value was associated with a significantly higher risk for NAFLD (P for trend <0.001; **Table 4**), after adjusting for age, sex, smoking, alcohol habitus and BMI. Further adjustment for duration of diabetes, family history for diabetes, hypertension, HbA1c, triglycerides, total cholesterol, and medications showed similar findings (P for trend <0.001), although the difference between TFQI Q2/Q3 and Q1 was not statistically significant. This association was slightly changed but statistically significant when the role of insulin resistance was examined (Q4 vs. Q1: OR 1.27, 95% CI 1.03-1.56, P =0.025; P for trend =0.017; **Table 4**).

**Table 4:** Associations between thyroid hormone sensitivity and NAFLD in hospitalized type 2 diabetic patients.

		Model 1		Model 2		Model 3		Model 4	
		OR (95% CI)	P value	OR (95% CI)	P value	OR (95% CI)	P value	OR (95% CI)	P value
TFQI	Quartile 1	1.00 (Reference)		1.00 (Reference)		1.00 (Reference)		1.00 (Reference)	
	Quartile 2	1.22 (1.07, 1.38)	0.002	1.19 (1.04, 1.36)	0.009	1.04 (0.89, 1.22)	0.624	0.91 (0.75, 1.11)	0.365
	Quartile 3	1.37 (1.21, 1.56)	<0.001	1.36 (1.19, 1.55)	<0.001	1.14 (0.97, 1.33)	0.113	1.00 (0.82, 1.22)	1.00
	Quartile 4	1.54 (1.36, 1.75)	<0.001	1.59 (1.38, 1.83)	<0.001	1.39 (1.18, 1.63)	<0.001	1.27 (1.03, 1.56)	0.025
	P value for trend		<0.001		<0.001		<0.001		0.017
TT4RI	Quartile 1	1.00 (Reference)		1.00 (Reference)		1.00 (Reference)		1.00 (Reference)	
	Quartile 2	1.36 (1.2, 1.54)	<0.001	1.36 (1.20, 1.55)	<0.001	1.23 (1.05, 1.44)	0.01	1.09 (0.89, 1.32)	0.416
	Quartile 3	1.32 (1.16, 1.5)	<0.001	1.33 (1.17, 1.52)	<0.001	1.19 (1.02, 1.4)	0.027	1.12 (0.92, 1.36)	0.271
	Quartile 4	1.56 (1.37, 1.77)	<0.001	1.59 (1.39, 1.81)	<0.001	1.28 (1.1, 1.5)	0.002	1.13 (0.92, 1.38)	0.26
	P value for trend		<0.001		<0.001		0.005		0.247
TSHI	Quartile 1	1.00 (Reference)		1.00 (Reference)		1.00 (Reference)		1.00 (Reference)	
	Quartile 2	1.34 (1.16, 1.54)	<0.001	1.31 (1.13, 1.51)	<0.001	1.13 (0.95, 1.35)	0.164	1.03 (0.83, 1.28)	0.794
	Quartile 3	1.56 (1.35, 1.82)	<0.001	1.52 (1.3, 1.78)	<0.001	1.22 (1.02, 1.47)	0.033	1.04 (0.82, 1.31)	0.762
	Quartile 4	1.74 (1.49, 2.02)	<0.001	1.79 (1.53, 2.11)	<0.001	1.43 (1.19, 1.72)	<0.001	1.25 (0.98, 1.59)	0.068
	P value for trend		<0.001		<0.001		<0.001		0.045

Model 1 is adjusted for age, sex; model 2 is adjusted for age, sex, smoking, alcohol habitus, and body mass index; model 3 is adjusted for age, sex, smoking, alcohol habitus, body mass index, duration of diabetes, family history for diabetes, hypertension, HbA1c, triglyceride, total cholesterol, and medications; model 4 is adjusted for age, sex, smoking, alcohol habitus, body mass index, duration of diabetes, family history for diabetes, hypertension, HbA1c, triglyceride, total cholesterol, medications, and HOMA-IR.

OR, odds ratio; TFQI, thyroid feedback quantile-based index; TSHI, TSH index; TT4RI, Thyrotropin T4 resistance index.

We further analyzed the association between these indices and NAFLD stratified by FIB-4 score. Multivariable-adjusted analysis revealed a significant association between TFQI and NAFLD with low FIB-4 scores (Q4 vs. Q1: OR 1.42, 95% CI 1.15–1.75, P=0.001) (P for trend =0.001), but not NAFLD with intermediate-to-high FIB-4 scores (P for trend =0.195) (**Table 5**).

**Table 5:** Associations between thyroid hormone sensitivity and NAFLD with low- or intermediate-to-high probability of advanced fibrosis in hospitalized type 2 diabetic patients.

		NAFLD with low probability of advanced fibrosis		NAFLD with intermediate-to-high probability of advanced fibrosis	
		OR (95% CI)	P value	OR (95% CI)	P value
TFQI	Quartile 1	1.00 (Reference)		1.00 (Reference)	
	Quartile 2	0.99 (0.8, 1.23)	0.944	0.86 (0.66, 1.11)	0.238
	Quartile 3	1.1 (0.89, 1.36)	0.376	0.78 (0.61, 1.01)	0.783
	Quartile 4	1.42 (1.15, 1.75)	0.001	0.86 (0.67, 1.1)	0.232
	P value for trend		0.001		0.195
TT4RI	Quartile 1	1.00 (Reference)		1.00 (Reference)	
	Quartile 2	1.07 (0.87, 1.32)	0.521	1.03 (0.8, 1.34)	0.802
	Quartile 3	1.12 (0.91, 1.38)	0.289	1.13 (0.88, 1.46)	0.343
	Quartile 4	1.17 (0.94, 1.44)	0.156	1.13 (0.88, 1.46)	0.344
	P value for trend		0.14		0.266
TSHI	Quartile 1	1.00 (Reference)		1.00 (Reference)	
	Quartile 2	0.99 (0.77, 1.25)	0.899	1.07 (0.79, 1.43)	0.679
	Quartile 3	1.05 (0.81, 1.35)	0.713	1.13 (0.83, 1.55)	0.429
	Quartile 4	1.29 (1.01, 1.66)	0.046	1.02 (0.75, 1.39)	0.906
	P value for trend		0.009		0.952

Adjusted for age, sex, smoking, alcohol habitus, body mass index, duration of diabetes, family history for diabetes, hypertension, HbA1c, triglyceride, total cholesterol, medications, and HOMA-IR.

FT3, free triiodothyronine; FT4, free thyroxine; OR, odds ratio; TFQI, thyroid feedback quantile-based index; TSHI, TSH index; TT4RI, Thyrotropin T4 resistance index.

Similar associations were detected for TT4RI and TSHI (P for trend =0.005 and <0.001, respectively) in the overall analyses (**Table 4**). The association remained significant for TSHI (P for trend =0.045) after further adjustment for HOMA-IR, but became nonstatistically significant for TT4RI (P for trend =0.247). Analysis of NAFLD stratified by FIB-4 score showed similar trend for TSHI as with TFQI, but not for TT4RI (**Table 5**).

### Prediction of advanced fibrosis in at-risk participants from the health-examination cohort

A total of 14,037 participants in the health examination cohort were considered at-risk and included in the analysis, in which 262 participants had a FIB-4 score >2.67. Among them, 11,756 participants were non-diabetic and 2,281 were diabetic. In the overall at-risk cohort, a higher TFQI quartile showed a trend towards lower risk for high-probability of advanced fibrosis after multivariate adjustment (P for trend =0.06; **Table 6**), but TT4RI and TSHI did not (P for trend =0.988 and 0.337 respectively). Stratified analysis based on diabetic status revealed that a higher TFQI value was associated with a significantly lower risk for high-probability of advanced fibrosis in the diabetic group (P for trend =0.018) but not the non-diabetic at-risk group (P for trend =0.377). TT4RI and TSHI did not show statistically significant associations with advanced fibrosis in either groups (**Table 6**).

**Table 6:** Predictors of thyroid hormone sensitivity for high probability of advanced fibrosis in the at-risk cohort from the health examination population.

		Overall at-risk cohort		Non-diabetic at-risk cohort		Diabetic at-risk cohort	
		OR (95% CI)	P value	OR (95% CI)	P value	OR (95% CI)	P value
TFQI	Quartile 1	1.00 (Reference)		1.00 (Reference)		1.00 (Reference)	
	Quartile 2	0.83 (0.56, 1.23)	0.344	0.93 (0.59, 1.44)	0.731	0.55 (0.22, 1.38)	0.204
	Quartile 3	0.8 (0.54, 1.19)	0.274	0.77 (0.48, 1.22)	0.257	0.95 (0.43, 2.12)	0.897
	Quartile 4	0.66 (0.43, 1.01)	0.055	0.86 (0.54, 1.37)	0.527	0.16 (0.04, 0.58)	0.005
	P value for trend		0.06		0.377		0.018
TT4RI	Quartile 1	1.00 (Reference)		1.00 (Reference)		1.00 (Reference)	
	Quartile 2	0.94 (0.61, 1.42)	0.75	0.88 (0.54, 1.43)	0.611	1.19 (0.5, 2.78)	0.697
	Quartile 3	0.85 (0.56, 1.29)	0.448	0.96 (0.6, 1.53)	0.862	0.54 (0.21, 1.41)	0.209
	Quartile 4	1.03 (0.69, 1.56)	0.875	1.24 (0.79, 1.96)	0.355	0.58 (0.18, 1.27)	0.137
	P value for trend		0.988		0.315		0.059
TSHI	Quartile 1	1.00 (Reference)		1.00 (Reference)		1.00 (Reference)	
	Quartile 2	0.73 (0.48, 1.1)	0.128	0.81 (0.51, 1.28)	0.36	0.51 (0.19, 1.32)	0.162
	Quartile 3	0.82 (0.55, 1.22)	0.331	0.86 (0.55, 1.36)	0.514	0.77 (0.33, 1.8)	0.549
	Quartile 4	0.79 (0.52, 1.18)	0.249	0.93 (0.59, 1.47)	0.759	0.4 (0.15, 1.03)	0.057
	P value for trend		0.337		0.814		0.107

Adjusted for age, sex, BMI, hypertension, HbA1c, triglyceride, and total cholesterol.

OR, odds ratio; TFQI, thyroid feedback quantile-based index; TSHI, TSH index; TT4RI, Thyrotropin T4 resistance index.

### Prediction of advanced fibrosis in the hospitalized type 2 diabetic cohort

All 8,246 patients contributed to the analysis, in which 821 patients showed a FIB-4 score >2.67, an incidence much higher than that in the health-examination cohort. Multivariate analysis showed that a higher TFQI value was associated with a significantly lower risk of high-probability of advanced fibrosis (P for trend <0.001; **Table 7**), after adjustment for multiple variables including HOMA-IR. TSHI showed similar association but the magnitude was weaker (P for trend =0.042). No association was detected between TT4RI and advanced fibrosis in this cohort. Consistent findings were detected using a low FIB-4 score as the outcome (**Table 7**).

**Table 7:** Predictors of thyroid hormone sensitivity for high and low probability of advanced fibrosis with FIB-4 score in hospitalized type 2 diabetic patients.

		High probability of advanced fibrosis		Low probability of advanced fibrosis	
		OR (95% CI)	P value	OR (95% CI)	P value
TFQI	Quartile 1	1.00 (Reference)		1.00 (Reference)	
	Quartile 2	1.01 (0.74, 1.37)	0.954	1.01 (0.83, 1.21)	0.99
	Quartile 3	0.63 (0.46, 0.88)	0.007	1.28 (1.05, 1.55)	0.013
	Quartile 4	0.62 (0.45, 0.86)	0.005	1.17 (0.97, 1.42)	0.1
	P value for trend		<0.001		0.019
TT4RI	Quartile 1	1.00 (Reference)		1.00 (Reference)	
	Quartile 2	0.96 (0.69, 1.33)	0.797	0.93 (0.76, 1.12)	0.436
	Quartile 3	0.93 (0.67, 1.28)	0.643	0.91 (0.75, 1.1)	0.346
	Quartile 4	0.86 (0.63, 1.19)	0.374	0.9 (0.74, 1.09)	0.267
	P value for trend		0.363		0.274
TSHI	Quartile 1	1.00 (Reference)		1.00 (Reference)	
	Quartile 2	1.05 (0.74, 1.49)	0.797	0.91 (0.73, 1.13)	0.383
	Quartile 3	0.72 (0.49, 1.06)	0.093	0.96 (0.77, 1.21)	0.961
	Quartile 4	0.8 (0.55, 1.16)	0.239	1.02 (0.82, 1.29)	0.84
	P value for trend		0.042		0.436

Adjusted for age, sex, BMI, smoking status, alcohol habitus, duration of diabetes, family history for diabetes, hypertension, HbA1c, triglyceride, total cholesterol, medications, and HOMA-IR.

OR, odds ratio; TFQI, thyroid feedback quantile-based index; TSHI, TSH index; TT4RI, Thyrotropin T4 resistance index.

## Discussion

In two large cohorts, our study showed for the first time that indices of TH sensitivity, particularly the newly developed TFQI, were associated with an increased risk of NAFLD in type 2 diabetic patients but not in non-diabetic participants. The increased NAFLD risk associated with higher TFQI in type 2 diabetic patients was limited to those with a low probability of advanced fibrosis, and was not observed in patients with an intermediate-to-high probability of advanced fibrosis. Furthermore, a higher TFQI was associated with a significantly lower risk of advanced fibrosis in diabetic at-risk patients, but this association was not observed in non-diabetic at-risk participants, independent of NAFLD status. These significant associations persisted after adjusting for multiple potential confounders, including HOMA-IR.

One preliminary study with 4,610 subjects from a health examination population revealed no significant association between TFQI and NAFLD development (14). Our study substantially extended this finding in several aspects. First, our study sample size was much larger than that of the previous study (26,413 vs 4,610), thus the power of the analysis increased as well. Second, we also performed analysis of other TH sensitivity indices such as TT4RI and TSHI, and showed similar findings to TFQI. Third, we confirmed the predictive role of diabetes on NAFLD, and for the first time performed a stratified analysis of the effect of TH sensitivity indices on NAFLD development based on diabetic status. Fourth, we evaluated the role of these indices in predicting advanced fibrosis, as represented by the FIB-4 score. All findings based on diabetic status analyses were novel in the literature, to the best of our knowledge.

The association between TH sensitivity indices and NAFLD provides a rational explanation for the aforementioned contradictory association of high THs or high TSH with NAFLD (7, 9). A higher TFQI profile, often characterized by both coincident high-normal TSH and FT4 levels, resembles that of resistance to TH (RTH) syndrome, an inherited rare disorder (20). Patients with RTH syndrome harbor mutations in the *THRB* gene, which leads to impaired negative regulation of the hypothalamus–pituitary–thyroid axis (20). All these evaluated index measure central resistance (pituitary gland) inhibition by FT4 levels (10); the proposal of the concept of acquired resistance to TH in the general population also provides some plausible biological mechanisms for the relationship between thyroid function and many other metabolic syndromes, including hyperuricemia, obesity, and cardiovascular disease, beyond NAFLD.

Recent research suggests that intrahepatic hypothyroidism, particularly reduced thyroid hormone signaling within liver tissues, may worsen lipid accumulation and fibrosis in NAFLD. Intrahepatic hypothyroidism could result from decreased production of active thyroid hormone (T3) and increased inactivation of thyroxine (T4) by specific enzymes in the liver, thus promoting metabolic and inflammatory disturbances in NAFLD. Liver-specific thyroid hormone receptor β1 (THR-β1) agonists, such as resmetirom, have shown promise in preclinical and clinical studies for reducing hepatic fat and inflammation in NAFLD without systemic side effects (21).

The observational design of our study does not allow establishing causal relationships between TH sensitivity and NAFLD, but a causal association is indicated based on findings from previously published clinical and experimental studies. Preclinical studies suggested that impaired autophagy, mitophagy, and mitochondrial function contributed to the pathogenesis of NAFLD (22).TH represents a compound capable of restoring these metabolic processes and may, therefore, serve as a promising target for drug discovery in the treatment of NAFLD. Indeed, TH and its analogs, such as resmetirom (MGL-3196), eprotirome, and sobetirome, prove to be effective in treating liver steatosis in preclinical models and patients with NAFLD (23–25). Specially, resmetirom (MGL-3196), an orally active, selective TH receptor β1 (THRβ1) agonist to treat obesity and hypercholesterolemia, reduced hepatic fat content at week 12, with a greater proportion of treated patients showing pathological improvement in liver biopsy samples (26). Additionally, endogenous thyroid hormone metabolites like 3,5-T2 have shown anti-steatotic effects by promoting fatty acid oxidation and reducing triglyceride accumulation in the liver (27).

A key finding of our study was that the predictive effect of TH sensitivity indices on NAFLD development was observed only in patients with type 2 diabetes and not in non-diabetic participants. Diabetes is a risk factor for NAFLD, with the prevalence of NAFLD in type 2 diabetic patients is reported to be 55.5% (28), much higher than that in the general population. In agreement, the incidence of NAFLD was 20.1% in the non-diabetic population, 33.8% in patients with diabetes from health screening programs, and 46.5% in patients hospitalized for diabetes in our cohorts (**Supplementary Table S1**).

Thyroid function is critical in regulating carbohydrate metabolism and pancreatic function, and thyroid dysfunction is also associated with additional increased risk of incident diabetes (29, 30). The interaction between thyroid dysfunction and diabetes is bidirectional, and L-thyroxine treatment may significantly improve insulin sensitivity in patients with hypothyroidism and insulin resistance, suggesting that modulating thyroid status could be a potential strategy for reducing NAFLD risk (31, 32). Given the close relationship between diabetes and thyroid dysfunction, a stronger relationship between thyroid function and NAFLD among patients with diabetes seems plausible. It’s notable that our findings were obtained in euthyroid patients, suggesting that reduced central TH sensitivity, even within the normal thyroid function range, is closely associated with NAFLD in individuals with diabetes. The association was attenuated but remained statistically significant when the insulin resistance index HOMA-IR was further adjusted, suggesting importantly that insulin resistance did not seem to fully explain the association between TH resistance and NAFLD in our study. Hypothyroidism may be linked to insulin resistance (33), which is also a common pathophysiological mechanism underlying both diabetes and NAFLD. In light of the shared pathogenic background, it may be plausible that thyroid dysfunction and diabetes have additive effect on NAFLD development. Other mechanisms are also involved in the association between thyroid and NAFLD in the context of diabetes. A detailed description of the potential multifactorial mechanisms involved in diabetes is beyond the scope of our analysis, however, relevant mechanisms may include extrahepatic factors, such as low-grade inflammation, and intrahepatic factors, such as increased oxidative stress, both of which have been shown to contribute to the development of NAFLD and other metabolic disorders (16, 34).

According to the recently published clinical care pathway from the American Gastroenterological Association, three types of people, i.e., those with two or more metabolic risk factors, with type 2 diabetes, or with steatosis on imaging or elevated aminotransferases, were at the greatest risk and therefore should be screened for NAFLD-related fibrosis (17). It’s interesting in our study that TH sensitivity indices, particularly TFQI-associated NAFLD risk increase in type 2 diabetic patients, was restricted to NAFLD with low probability of advanced fibrosis, but not applied to those with intermediate-to-high fibrosis probability, as indicated by non-invasive FIB-4 score. TH sensitivity was associated with the development of NAFLD in our diabetic population; however, it did not correlate with the severity of NAFLD. In contrast, in diabetic at-risk patients, a higher TFQI was associated with a significantly lower risk of having a high probability of advanced fibrosis, independent of NAFLD status; this association was not observed in non-diabetic at-risk participants.

Kim and colleagues reported that “low-normal” thyroid function, defined as a TSH level between 2.5 and 4.5 mIU/L and normal free T4 level, was associated with a higher risk of fibrosis (35, 36).In our study, we performed a similar analysis based on this definition and confirmed a positive association between low-normal thyroid function with advanced fibrosis defined on FIB-4 score, as compared with strict-normal thyroid function in diabetic individuals (OR 1.17, 95% CI 1.02-1.35, P=0.03). The discrepancy between analysis on regular thyroid function parameters and TH sensitivity parameters suggests different interpretations of these parameters are needed. Our findings indicate that TH sensitivity indices differ significantly in predicting NAFLD development and advanced fibrosis between individuals with and without type 2 diabetes. This suggests that future studies on NAFLD should consider stratified analyses or trials based on diabetic status. Given that NAFLD pathogenesis may differ between diabetic and non-diabetic populations, conducting such studies could enhance the precision of NAFLD treatments and facilitate more targeted therapeutic approaches. This approach is particularly crucial, as there are currently no approved treatments for NAFLD, which may be partly attributable to the heterogeneous nature of its pathogenesis (37). Our study also suggests that it might be reasonable for individuals with type 2 diabetes to undergo thyroid function tests, to help determine the risk of NAFLD development and advance fibrosis. However, the predictive effect of TH sensitivity indices on advanced fibrosis warrants confirmation in well-organized prospective studies in patients with biopsy data.

Our study has several strengths: the large sample size, the validation of diabetic findings in two separate cohorts, the inclusion of a wealth of demographic and metabolic variables for multivariate adjustment, the assessment of three different indices of TH resistance for complementary, etc. Nonetheless, it is important to acknowledge a few limitations. First, our study shared the inherent limitations of retrospective studies including data missing, although the proportion was low and can be partially compensated by the large cohort size. Second, NAFLD was diagnosed through ultrasound examination in our study, which is not sensitive enough to detect mild steatosis, and might subject to interobserver and intraobserver diagnostic variability.even though all ultrasonographic examinations were performed by experienced radiologists. Third, due to the cross-sectional study design, a causal relationship could not be documented by this study, and further evidence is needed. Fourth, we did not collect quantitative data on alcohol intake and therefore adjusted for alcohol consumption as a dichotomous variable (drinker vs. non-drinker). However, we believe this approach would not significantly impact the findings, as excessive alcohol consumption is uncommon in the Chinese population (3, 38). Finally, our study used the non-invasive FIB-4 score as a surrogate marker of fibrosis. Although FIB-4 have been widely used and well validated by liver biopsy (39), future studies with histological data are needed.

## Conclusions

Our study showed that TH resistance was associated with an increased risk of developing NAFLD but was associated with a reduced risk of advanced fibrosis in diabetic individuals. Our findings suggest that stratified studies of NAFLD based on diabetic status are needed in future.

## Data Availability

The raw data supporting the conclusions of this article will be made available by the authors, without undue reservation.
