# Supplementary material for: Impaired sensitivity to thyroid hormone is associated with developing non-alcoholic fatty liver disease in euthyroid diabetic subjects
Source: Front Endocrinol (Lausanne). 2024 Dec 18;15:1450049. doi: 10.3389/fendo.2024.1450049 (PMC11688208; doi:10.3389/fendo.2024.1450049)
Supplement: Supplementary file 1 [file DataSheet1.docx]

**Supplementary materials**

**
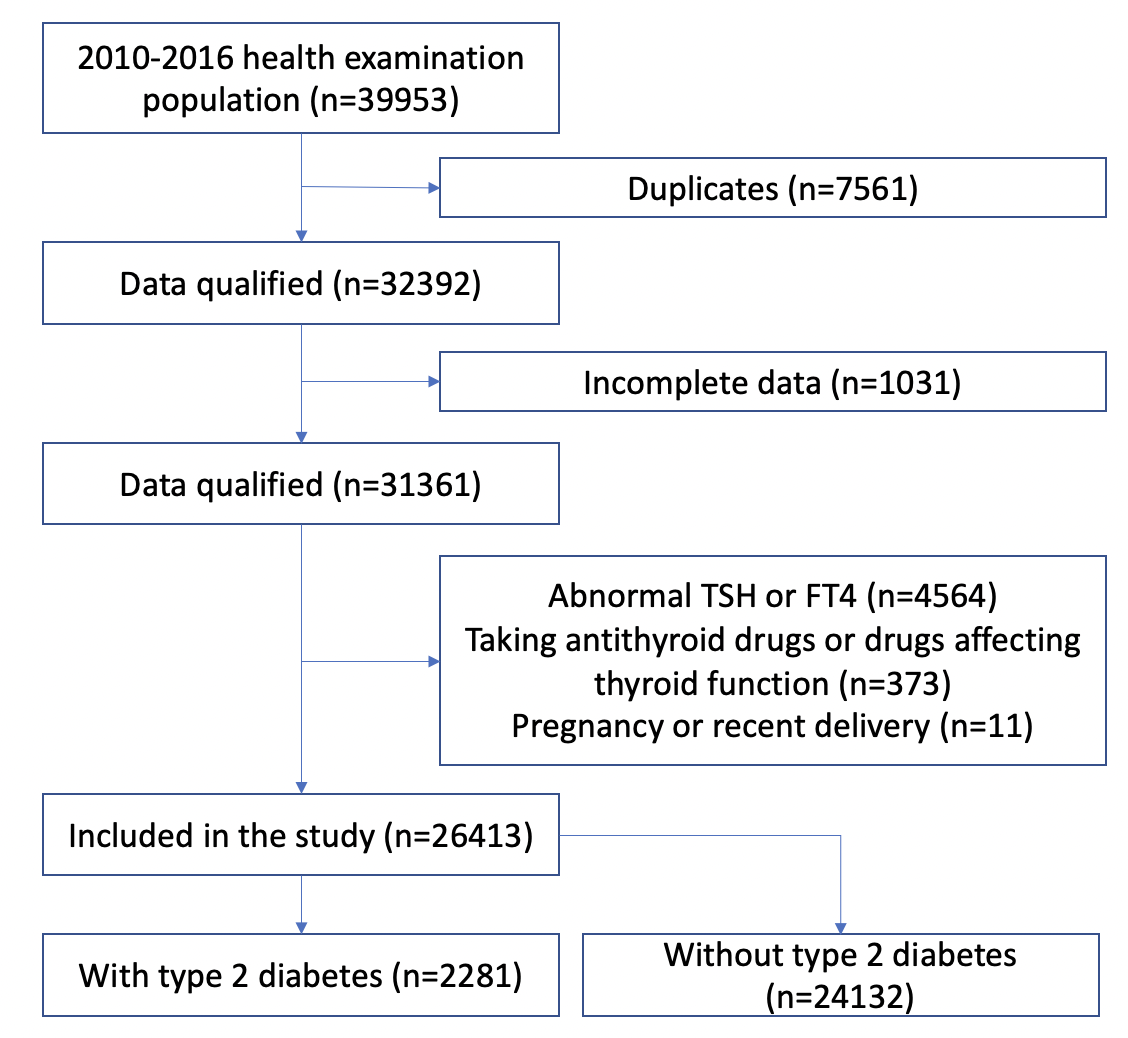
**

**Figure S1.** flow chart of the study participants in the health examination population.

**
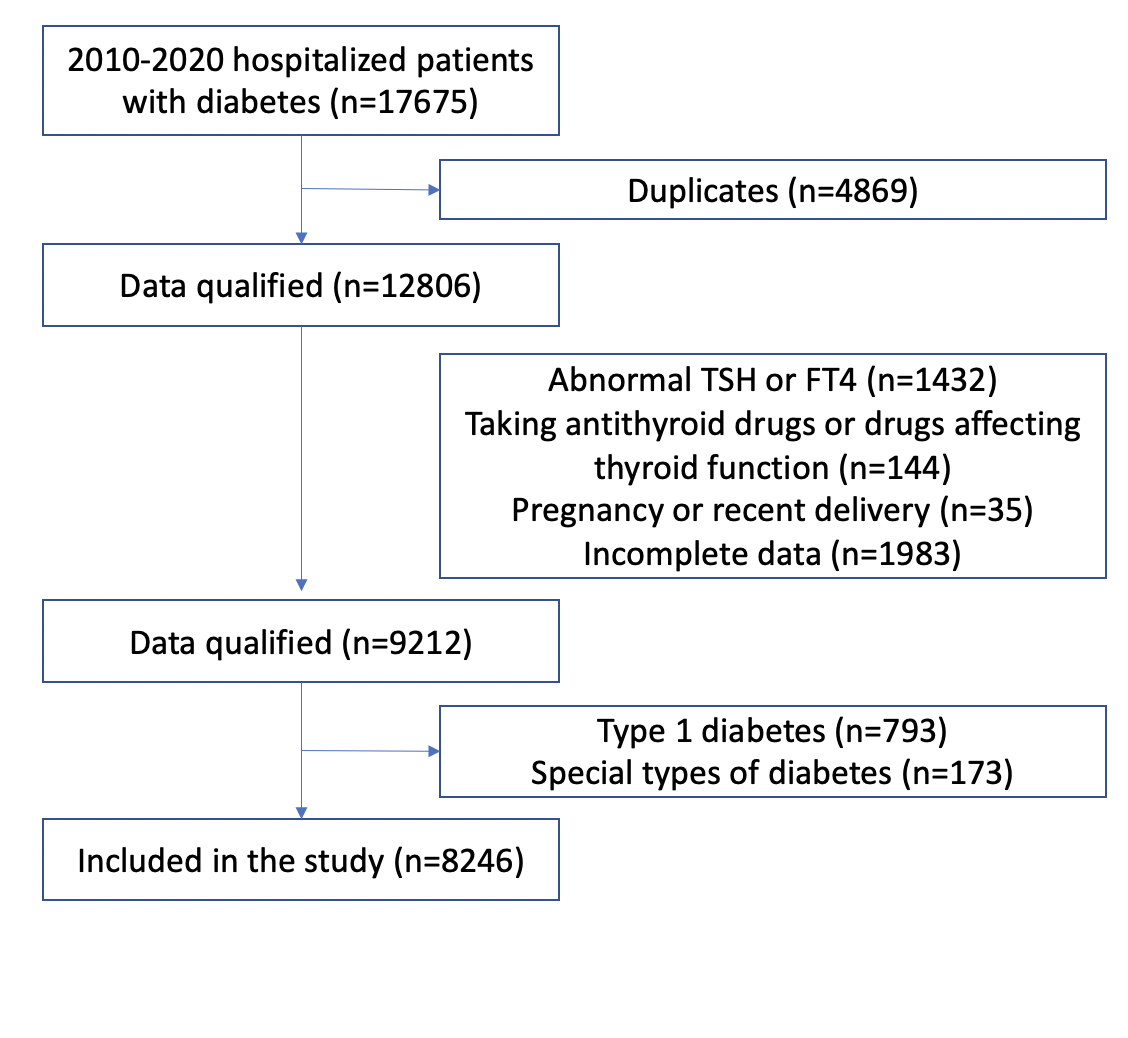
**

**Figure S2.** flow chart of the hospitalized patients with type 2 diabetes.

**Table S1.** Baseline characteristics of the health examination population according to diabetic status.

|  | Overall | Diabetic population | Non-diabetic population | P value |
| --- | --- | --- | --- | --- |
| N | 26413 | 2281 | 24132 |  |
| Age (ys) | 48.34±15.22 | 48.36±15.34 | 48.34±15.2 | 0.949 |
| Sex, male | 15247 (57.7%) | 1386 (60.8%) | 13861 (57.4%) | 0.002 |
| BMI | 24.22±3.17 | 24.16±3.15 | 24.22±3.18 | 0.375 |
| SBP (mmHg) | 125.75±17.71 | 126.05±17.52 | 125.72±17.73 | 0.414 |
| HbA1c (%) | 5.7±0.81 | 7.1±1.39 | 5.5±0.36 | <0.001 |
| FBG (mmol/L) | 5.36±1.14 | 7.75±2.32 | 5.13±0.56 | <0.001 |
| TG (mmol/L) | 1.55±1.26 | 2.1±1.86 | 1.49±1.17 | <0.001 |
| TC (mmol/L) | 4.84±0.91 | 4.92±1.04 | 4.83±0.89 | <0.001 |
| LDL-C (mmol/L) | 2.57±0.69 | 2.62±0.78 | 2.56±0.68 | <0.001 |
| HDL-C (mmol/L) | 1.3±0.36 | 1.17±0.33 | 1.31±0.36 | <0.001 |
| ALT (IU/L) | 25.61±19.83 | 30.42±21.29 | 25.15±19.62 | <0.001 |
| AST (IU/L) | 22.42±9.98 | 24±11.41 | 22.27±9.82 | <0.001 |
| Platelet count (×10^9^/L) | 220.21±53.01 | 208.32±52.39 | 221.35±52.93 | <0.001 |
| Albumin (g/L) | 44.42±2.47 | 44.18±2.61 | 44.44±2.46 | <0.001 |
| Creatine (μmol/L) | 68.23±143.88 | 70.07±18.35 | 68.05±150.57 | 0.523 |
| TSH (pmol/L) | 2.17±0.86 | 2.21±0.89 | 2.16±0.86 | 0.015 |
| FT3 (pmol/L) | 4.9±0.67 | 4.78±0.67 | 4.91±0.67 | <0.001 |
| FT4 (pmol/L) | 16.94±2.1 | 17.03±2.09 | 16.94±2.1 | 0.034 |
| NAFLD | 5640 (21.4%) | 772 (33.8%) | 4868 (20.1%) | <0.001 |
| Hypertension | 5481 (20.8%) | 460 (20.2%) | 5021 (20.8%) | 0.471 |

Continuous variables are presented as mean ± standard deviation (SD) or median (interquartile range). Categorical variables are presented as number (percentage).

Abbreviations: ALT, alanine transaminase; AST, aspartate aminotransferase; BMI, body mass index; FBG, fasting blood glucose level; FT3, free triiodothyronine; FT4, free thyroxine; HDL-C, high-density lipoprotein cholesterol; LDL-C, low-density lipoprotein cholesterol; NAFLD, non-alcoholic fatty liver; SBP, systolic blood pressure; TC, total cholesterol; TG, triglycerides; TSH, thyroid stimulating hormone.

**Table S2.** Baseline characteristics of the non-diabetic health examination population according to TFQI categories.

|  | Total  (-1.0, 0.94) | Quartile 1  (-1.0, -0.25) | Quartile 2  (-0.25, 0.01) | Quartile 3  (0.01, 0.27) | Quartile 4  (0.27, 0.94) | P value |
| --- | --- | --- | --- | --- | --- | --- |
| N | 24132 | 6088 | 6052 | 6007 | 5985 |  |
| Age (ys) | 48.34±15.2 | 48.21±15.03 | 48.5±15.35 | 48.61±15.45 | 48.03±14.98 | 0.134 |
| Sex, male | 13861 (57.4%) | 3505 (57.6%) | 3454 (57.1%) | 3473 (57.8%) | 3429 (57.3%) | 0.855 |
| BMI | 24.22±3.18 | 24.26±3.18 | 24.23±3.22 | 24.24±3.18 | 24.15±3.13 | 0.256 |
| SBP (mmHg) | 125.72±17.73 | 125.55±17.64 | 125.87±17.67 | 126.04±17.97 | 125.42±17.64 | 0.226 |
| HbA1c (%) | 5.5±0.36 | 5.52±0.36 | 5.5±0.37 | 5.49±0.37 | 5.47±0.36 | <0.001 |
| FPG (mmol/L) | 5.13±0.56 | 5.13±0.56 | 5.13±0.55 | 5.12±0.56 | 5.13±0.55 | 0.384 |
| TG (mmol/L) | 1.49±1.17 | 1.5±1.4 | 1.5±1.18 | 1.5±1.08 | 1.48±1 | 0.632 |
| TC (mmol/L) | 4.83±0.89 | 4.83±0.92 | 4.83±0.89 | 4.84±0.88 | 4.83±0.88 | 0.821 |
| LDL-C (mmol/L) | 2.56±0.68 | 2.55±0.7 | 2.56±0.68 | 2.58±0.68 | 2.57±0.67 | 0.076 |
| HDL-C (mmol/L) | 1.31±0.36 | 1.32±0.37 | 1.31±0.36 | 1.31±0.36 | 1.3±0.36 | 0.264 |
| ALT (IU/L) | 25.15±19.62 | 24.97±18.42 | 24.91±20.58 | 25.2±20.94 | 25.52±18.4 | 0.311 |
| AST (IU/L) | 22.27±9.82 | 22.12±9.06 | 22.24±10.37 | 22.3±9.96 | 22.4±9.85 | 0.494 |
| Platelet count (×10^9^/L) | 221.35±52.93 | 221.91±54.23 | 221.17±53.29 | 221.4±52.32 | 220.9±51.85 | 0.763 |
| Albumin (g/L) | 44.44±2.46 | 44.15±2.44 | 44.36±2.42 | 44.49±2.43 | 44.79±2.49 | <0.001 |
| Creatinine (μmol/L) | 68.05±150.57 | 64.76±23.63 | 66.42±19.98 | 71.96±299.44 | 69.1±16.24 | 0.051 |
| TSH (pmol/L) | 2.16±0.86 | 1.44±0.47 | 2.02±0.8 | 2.37±0.82 | 2.83±0.66 | <0.001 |
| FT3 (pmol/L) | 4.91±0.67 | 4.83±0.73 | 4.88±0.65 | 4.93±0.63 | 4.99±0.64 | <0.001 |
| FT4 (pmol/L) | 16.94±2.1 | 15.3±1.45 | 16.34±1.98 | 17.37±1.76 | 18.76±1.39 | <0.001 |
| NAFLD | 4868 (20.2%) | 1276 (21.0%) | 1224 (20.2%) | 1169 (19.5%) | 1199 (20.0%) | 0.23 |
| Hypertension | 5021 (20.8%) | 1314 (21.6%) | 1275 (21.7%) | 1239 (20.6%) | 1193 (19.9%) | 0.147 |

Continuous variables are presented as mean ± standard deviation (SD) or median (interquartile range). Categorical variables are presented as number (percentage).

Abbreviations: ALT, alanine transaminase; AST, aspartate aminotransferase; BMI, body mass index; FPG, fasting plasma glucose; FT3, free triiodothyronine; FT4, free thyroxine; HDL-C, high-density lipoprotein cholesterol; LDL-C, low-density lipoprotein cholesterol; NAFLD, non-alcoholic fatty liver disease; SBP, systolic blood pressure; TC, total cholesterol; TFQI, thyroid feedback quantile-based index; TG, triglycerides; TSH, thyroid stimulating hormone.

**Table S3.** Associations between thyroid hormone sensitivity and NAFLD in type 2 diabetic health examination population.

|  |  | Model 1 |  | Model 2 |  | Model 3 |  |
| --- | --- | --- | --- | --- | --- | --- | --- |
|  |  | OR (95% CI) | P value | OR (95% CI) | P value | OR (95% CI) | P value |
| TFQI | Quartile 1 | 1.00 (Reference) |  | 1.00 (Reference) |  | 1.00 (Reference) |  |
|  | Quartile 2 | 1.36 (1.04, 1.78) | 0.027 | 1.4 (1.05, 1.87) | 0.021 | 1.34 (0.996, 1.81) | 0.053 |
|  | Quartile 3 | 2.05 (1.58, 2.66) | <0.001 | 2.24 (1.69, 2.96) | <0.001 | 2.12 (1.59, 2.82) | <0.001 |
|  | Quartile 4 | 1.89 (1.46, 2.45) | <0.001 | 1.84 (1.39, 2.43) | <0.001 | 1.82 (1.36, 2.42) | <0.001 |
|  | P value for trend | | <0.001 |  | <0.001 |  | <0.001 |
| TT4RI | Quartile 1 | 1.00 (Reference) |  | 1.00 (Reference) |  | 1.00 (Reference) |  |
|  | Quartile 2 | 1.08 (0.83, 1.39) | 0.583 | 1.07 (0.82, 1.42) | 0.614 | 1.004 (0.75, 1.34) | 0.98 |
|  | Quartile 3 | 1.29 (0.997, 1.66) | 0.052 | 1.21 (0.92, 1.58) | 0.172 | 1.14 (0.86, 1.51) | 0.359 |
|  | Quartile 4 | 1.36 (1.06, 1.74) | 0.014 | 1.33 (1.02, 1.74) | 0.033 | 1.31 (0.998, 1.73) | 0.051 |
|  | P value for trend | | 0.006 |  | 0.021 |  | 0.031 |
| TSHI | Quartile 1 | 1.00 (Reference) |  | 1.00 (Reference) |  | 1.00 (Reference) |  |
|  | Quartile 2 | 1.23 (0.94, 1.6) | 0.133 | 1.2 (0.91, 1.6) | 0.198 | 1.15 (0.86, 1.54) | 0.35 |
|  | Quartile 3 | 1.6 (1.24, 2.06) | <0.001 | 1.61 (1.22, 2.11) | 0.001 | 1.55 (1.17, 2.04) | 0.002 |
|  | Quartile 4 | 1.61 (1.25, 2.07) | <0.001 | 1.54 (1.18, 2.01) | 0.002 | 1.52 (1.15, 2.01) | 0.003 |
|  | P value for trend | | <0.001 |  | <0.001 |  | 0.001 |

Model 1 is adjusted for age, sex; model 2 is adjusted for age, sex, and BMI; model 3 is adjusted for age, sex, BMI, hypertension, HbA1c, triglyceride, and total cholesterol.

Abbreviations: FT3, free triiodothyronine; FT4, free thyroxine; OR, odds ratio; TFQI, thyroid feedback quantile-based index; TSHI, TSH index; TT4RI, Thyrotropin T4 resistance index.
